# Brain Delivery of Antibody-Derived Biologicals for Alzheimer’s Disease: An Updated Narrative Review

**DOI:** 10.3390/antib15020037

**Published:** 2026-04-17

**Authors:** Rachita K. Sumbria, Ruben J. Boado

**Affiliations:** 1 Department of Biomedical and Pharmaceutical Sciences and Center for Targeted Drug Delivery (CTDD), School of Pharmacy, Chapman University, Irvine, CA 92618, USA; sumbria@chapman.edu; 2 Department of Neurology, University of California, Irvine, CA 92697, USA; 3 Department of Medicine, University of California, Los Angeles (UCLA), Los Angeles, CA 90095, USA

**Keywords:** Alzheimer’s disease, bispecific antibodies, blood–brain barrier, decoy receptors, neurotrophins, protein-based therapy, receptor-mediated transcytosis, transferrin receptor

## Abstract

Antibodies directed against β-amyloid (Aβ) have been developed for the treatment of Alzheimer’s disease (AD). However, the in vivo central efficacy is reduced by the poor penetration of antibodies across the blood–brain barrier (BBB). In addition, these antibodies have been associated with adverse effects like amyloid-related imaging abnormalities. Thus, the development of new antibody-based therapies for AD with improved transport across the BBB may improve efficacy and reduce adverse effects. Antibodies targeting the BBB transferrin receptor (TfR) are able to cross the BBB through receptor-mediated transcytosis, producing a global distribution throughout the brain. Along the same line, bispecific antibodies directed to both the BBB TfR and Aβ showed enhanced brain uptake and pharmacological effects with diminished adverse side effects in experimental animal models of AD and in clinical trials. A generation of brain-penetrating fusion proteins targeting the BBB-TfR has been shown to represent novel treatments for AD, and this includes erythropoietin, tumor necrosis factor alpha inhibitors, neprilysin, somatostatin, oligonucleotides, and an antibody activating TREM2. The aim of this article is to review the progress made in the delivery of antibody-derived biologicals to the brain for AD, targeting the BBB-TfR.

## 1. Introduction

The immunotherapy of Alzheimer’s disease (AD) with antibodies directed against β-amyloid (Aβ) has been proposed as a potential treatment for this central nervous system (CNS) disorder, and several antibodies targeting Aβ have been approved by regulatory agencies in the USA and in the EU, i.e., aducanumab, donanemab, and lecanemab [1,2,3,4,5,6]. The latter are unmodified full IgG aimed at reducing Aβ fibrils and/or plaques in the brain to slow cognitive decline [1,2,3,4,5,6]. However, due to the presence of the blood–brain barrier (BBB), the brain penetration of these antibodies is markedly reduced, and their marginal therapeutic efficacy is associated with adverse effects like amyloid-related imaging abnormalities (ARIA) [7]. Therefore, there is a critical need for the development of new therapies for AD with improved transport across the BBB and reduced adverse effects.

The BBB is permeable to lipophilic small molecules of <400 Da, and proteins, in general, do not cross this barrier [8,9,10]. However, few proteins, like insulin, transferrin, insulin-like growth factor 1 (IGF1), and leptin, are able to cross the BBB via specific transport systems that induce receptor-mediated transcytosis (RMT) across the brain microvasculature forming the BBB [8,9,10]. Based on this finding, it was postulated that molecular Trojan horses and/or shuttle vectors may be developed to transport biologicals across the BBB by targeting these RMT systems [10,11]. Various monoclonal antibodies (MAbs) against these BBB receptors were developed, and those were able to penetrate the BBB without blocking the transport of their specific endogenous ligands, to produce brain levels of therapeutic relevance [12]. Using brain-penetrating MAbs, fusion proteins were produced and validated pre-clinically in animal models of AD, stroke, Parkinson’s disease, and lysosomal storage disorders [12]. Trontinemab is a bispecific MAb (BSA) directed against Aβ that crosses the BBB via the transferrin receptor (TfR), and is the first in this class to be evaluated in clinical trials of AD [13]. Other brain-penetrating BSAs in different formats are also under development, as well as fusion proteins aimed at treating AD by different mechanisms.

Given the rapidly emerging developments in the field of brain-penetrating antibody-based biologicals for AD, the purpose of this article is to provide a narrative review of the progress made in the advancement of treatments for AD targeting the BBB-TfR, including immunotherapy, neurotrophins, decoy receptors, and metalloproteases.

## 2. Erythropoietin for AD

**Figure 1:**
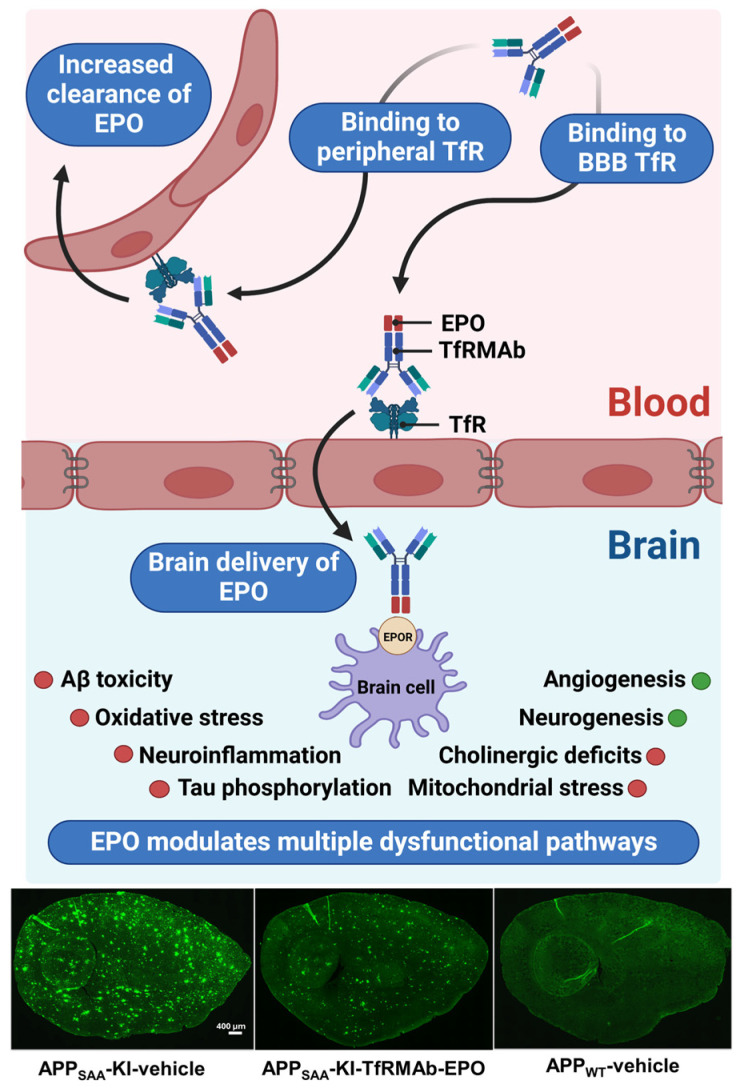
(**Top**): EPO fused to a molecular Trojan horse, in this case the transferrin receptor antibody (TfRMAb), is a BBB-penetrating EPO wherein the TfRMAb transports EPO into the brain across the BBB by receptor-mediated transport. In the periphery, TfRMAb-EPO binds to TfR on peripheral organs to facilitate faster plasma clearance compared to EPO alone, which reduces the plasma residence time and systemic side effects of TfRMAb-EPO. Once in the brain, the EPO domain of TfRMAb-EPO can bind to EPO receptors (EPORs) on brain cells to modulate multiple pathways involved in AD. Red dots represent a reduction by EPO, and green dots indicate an increase by EPO. (**Bottom**): Sagittal brain sections from male APP_SAA_ knock-in (KI) mice treated with either vehicle or TfRMAb-EPO (1 mg/kg) subcutaneously for 14 weeks show a robust reduction in 6E10-positive Aβ load. APP_WT_ mice, without the SAA mutations, are the genotype control and do not show Aβ deposits, as expected. From reference [31]. Abbreviations: APP_SAA_: amyloid precursor protein with the Swedish, Arctic, and Austrian mutations; WT: wild-type.

Erythropoietin (EPO) is a glycoprotein hormone that stimulates red blood cell (RBC) production and has been used to treat anemia since the 1980s [14]. Though EPO is primarily secreted from the kidneys, EPO and its receptors (EPORs) are also expressed by different brain cells [14], suggesting a non-hematopoietic role for EPO. Accordingly, today there is compelling evidence, both clinical and experimental, showing the neuroprotective effects of EPO. EPO has been shown to reduce toxicity associated with the major pathological hallmarks of AD, including Aβ and tau hyperphosphorylation, and stimulate endogenous neuroprotective and neuroregenerative pathways [15]. For example, in vitro, EPO reduced Aβ-induced cell death [16,17,18,19], and in rodent AD models, EPO reduced cognitive decline [20,21,22,23], endothelial damage [21], Aβ burden [23], tau hyperphosphorylation [24], cholinergic deficits [25], mitochondrial dysfunction and apoptosis [20], neuronal loss [26], and neuroinflammation [26], and enhanced neurogenesis [27] (Figure 1). The ability of EPO to reduce AD pathological hallmarks and promote neuroprotection and neurogenesis can delay or prevent new brain insult from occurring, and potentially reverse neuronal damage and cognitive decline in AD. Notably, brain EPO is reduced [28], whereas brain EPOR is increased in rodent AD models [28] and AD patients [29]. Further, neuroprotection by EPO is a function of EPOR expression [30], and increased EPOR expression in the AD brain is therefore expected to increase its neuroprotective effects, provided sufficient levels of EPO are maintained in the brain. Taken together, EPO can modulate many aberrant pathways, including neuroprotective and neurodegenerative pathways, making it a promising disease-modifying treatment for AD.

To be used as a CNS therapeutic, EPO must reach the brain. However, EPO is a large polar molecule, with a molecular weight of 30kDa, that cannot freely diffuse across the BBB [32]. The idea that EPO crosses the intact BBB via EPOR was based on one study that utilized biotinylated EPO in mice and showed biotin immunoreactivity surrounding brain capillaries [33]. However, the source of biotin (EPO-bound or free biotin that can cross the BBB [34]) was not confirmed. Two studies using radiolabeled EPO show that only 0.05–0.1% of exogenous systemic EPO crosses the intact BBB in rodents and primates [32,35]. Brain uptake of systemic EPO correlates with BBB disruption [36]; however, BBB disruption in AD patients may be heterogeneous and transient [37], making it an unreliable route for global brain drug delivery for AD. Therefore, the primary obstacle to the clinical development of EPO for AD is its limited BBB permeability.

To enhance brain drug delivery, EPO is administered using non-physiological approaches that alter the BBB [38] or at high systemic doses. EPO doses used in completed, ongoing, or recruiting clinical trials for brain diseases (i.e., 30,000–48,000 IU per dose) (ClinicalTrails.gov database) [39,40,41,42,43] are much higher than those used for chronic kidney disease (CKD)-associated anemia in humans (~100 IU/kg or ~7000 IU per dose) [39,40,41,42,43] to offset the limited BBB penetration of EPO [32]. However, high systemic EPO doses induce hematopoietic adverse effects [44,45]. Consequently, one of the first EPO clinical trials for stroke raised safety concerns, despite signs of neuroprotection [46], and high-dose EPO increased thrombosis in traumatic brain injury patients [41]. Further, EPO doses in experimental AD studies range from 500 to 5000 IU/kg [20,21,22,23,25,27,47,48,49], with 5000 IU/kg being the most widely used given the low brain uptake of EPO [32]. Among these, only two reported hematology indices and showed elevated hematocrit with chronic systemic EPO dosing [22,23]. Similarly, a 4000 IU/kg chronic dose of EPO increased hematocrit by 50–70% in mice [50]. Hematopoietic effects are less likely in short-term studies, but are relevant to AD, which requires long-term treatment. Alternate non-hematopoietic variants of EPO face similar challenges due to their large molecular weight [23,47,51], and consequently require large systemic doses [23] or are administered intranasally to bypass the BBB [52]. Therefore, despite the continued clinical interest in EPO as a CNS therapeutic, the adverse hematopoiesis resulting from chronic high-dose EPO limits its clinical translation for AD.

### 2.1. Receptor-Mediated Brain Delivery of EPO

To facilitate non-invasive brain delivery of EPO, human EPO was fused to the carboxyl terminal of the heavy chain of a high-affinity bivalent rat/mouse chimeric TfRMAb [53]. The fusion protein (TfRMAb-EPO), which is specific for the mouse TfR, offers key advantages: (a) TfRMAb binds to the luminal BBB TfR to enable non-invasive BBB penetration of EPO; (b) TfRMAb also binds to the peripheral TfR, allowing faster clearance and low plasma circulation time of TfRMAb-EPO (Figure 1) [53]. This is expected to reduce the adverse effects of chronic EPO dosing [54,55]. Accordingly, the plasma clearance of TfRMAb-EPO is ~10-times faster than that of EPO [53,56]. The brain uptake of TfRMAb-EPO is 2% injected dose (ID)/g brain in mice post intravenous (IV) injection [53], which is 30-fold higher than that of a non-targeting antibody [57]. The brain concentration of EPO 60 minutes after an IV dose of 110 µg/kg body weight was reported to be 12 ng/g brain [53]. TfRMAb-EPO transcytosis into the brain was confirmed by capillary depletion, and ~70% of TfRMAb-EPO was in the post-vascular compartment [53]. Fusion of EPO to TfRMAb did not alter the binding properties of either domain, as TfRMAb-EPO retained high-affinity binding to both TfR and EPOR, with a dissociation constant (KD) of approximately 0.05 nM [58].

### 2.2. TfRMAb-EPO in AD Mouse Models

TfRMAb-EPO has shown therapeutic efficacy in several AD mouse models. In 5.5-month-old APP/PS1 male mice, which co-express the mouse/human amyloid precursor protein (APP) with the Swedish mutation and a mutant human presenilin 1 (PS1), chronic intraperitoneal (IP) TfRMAb-EPO dosing reduced Aβ load, microgliosis, spatial reference memory deficits, and neuronal loss [59]. In older 9.5-month-old APP/PS1 AD male mice, TfRMAb-EPO reduced Aβ42 and memory deficits, and increased synaptophysin [60]. Notably, equimolar EPO alone did not result in these therapeutic effects [60]. Furthermore, chronic subcutaneous (SQ) dosing of TfRMAb-EPO led to a robust 70% reduction in brain Aβ load (Figure 1), accompanied by improved spatial reference memory in male knock-in AD mice, which carry humanizing Aβ mutations along with the Swedish, Arctic, and Austrian (SAA) mutations in the mouse App gene (APP_SAA_ KI mice) [31]. Apart from Aβ-lowering effects, chronic IP TfRMAb-EPO reduced phosphorylated tau (pTau), microgliosis, and abnormal behavioral phenotype in 6-month-old male and female mice, which overexpress mutant human microtubule-associated protein tau (PS19 mice) [61].

With respect to hematological changes with chronic dosing, TfRMAb-EPO resulted in stable RBC indices following 6-week chronic 3 mg/kg SQ dosing in APP/PS1 mice; equimolar EPO alone altered hematology indices [60]. Dose escalation from 1 to 6 mg/kg caused no change in hematocrit following 4-week SQ TfRMAb-EPO dosing to C57 male mice. A high dose of 20 mg/kg TfRMAb-EPO, however, reduced hematocrit, an effect most likely associated with the TfRMAb domain of the fusion protein [31]. Notably, adverse effects associated with the high-dose TfRMAb-EPO dosing were reversed after cessation of dosing [31]. The immune (anti-drug antibody, ADA) response to TfRMAb-EPO following chronic 1 mg/kg IV 3-week systemic dosing was low titer [62], and was comparable to the ADA response observed with a 20-fold higher SQ TfRMAb-EPO chronic dose [31]. Finally, chronic TfRMAb-EPO did not result in cerebral microhemorrhages [59], a major adverse response associated with the use of the current Aβ-lowering antibodies [63].

## 3. Biologic TNF-α Inhibitors for AD

Chronic peripheral and brain inflammation, once considered a consequence of AD, is now widely recognized as a therapeutically significant feature of AD [64,65,66]. However, current approaches to reduce inflammation for AD largely target either peripheral or brain inflammation, and have been unsuccessful so far, perhaps due to inappropriate time of treatment, side-effects, lack of brain penetration, or targeting a single compartment (peripheral vs. brain) [67,68,69] (also see NCT05744401 and NCT05450549). Tumor necrosis factor alpha (TNF-α), a major driver of peripheral and brain inflammation, mediates multiple pathological processes underlying AD [70], making TNF-α inhibitors (TNFIs) promising therapeutic candidates for AD. However, existing FDA-approved biologic TNFIs, the most potent TNFIs, can engage peripheral but not brain TNF-α, since these biologics do not cross the BBB [71], and are therefore delivered invasively in AD [72,73,74]. To target both peripheral and central TNF-α for AD, biologic TNFIs must be re-engineered for BBB penetration. Accordingly, XPro1595, which targets both peripheral and brain soluble TNF-α [75], has shown promising results in Aβ-positive individuals in a Phase 2 clinical trial (NCT05318976).

### 3.1. Receptor-Mediated Brain Delivery of Biologic TNFIs

Receptor-mediated transport systems, including TfR, can be leveraged for non-invasive brain delivery of macromolecules without requiring high or invasive doses [10]. Accordingly, a BBB-penetrable biologic TNFI, specific for the mouse, was engineered by fusing the amino terminus of the extracellular domain of the type-II human TNF receptor (decoy TNFR) to the carboxyl terminus of the heavy chain of a high-affinity bivalently chimeric TfRMAb to enable BBB penetration using RMT [76]. The fusion protein is designated TfRMAb-TNFR.

TfRMAb-TNFR (BBB-penetrating biologic TNFI) and etanercept (extracellular domain of type II TNFR fused to human IgG1 fragment crystallizable (Fc) domain; non-BBB penetrating biologic TNFI) bind TNF-α with high-affinity (KD values of 374 ± 77 and 280 ± 80 pM, respectively) [77]. TfRMAb-TNFR rapidly enters the mouse brain after IV administration with a brain uptake of ~3% ID/g brain, which is more than 45-fold higher than the brain uptake of control IgG [76]. Based on this brain uptake, TNFR brain levels of 65 ng/g were achieved 60 minutes following the IV dosing of 350 µg/kg body weight of the fusion protein [76]. Successful transcytosis across the BBB was confirmed using the capillary depletion technique, which showed that 76% of TfRMAb-TNFR was within the brain parenchyma [76]. A systemic and brain pharmacokinetic (PK) analysis of TfRMAb-TNFR for doses between 0.3 and 10 mg/kg in mice comparing the IP, SQ, and IV routes was performed, since extravascular routes are preferred over the IV route for a chronic disease like AD [78]. SQ and IP administration resulted in brain concentrations of TfRMAb-TNFR that were many-fold greater than pathologic neural TNF-α levels. E.g., peak TNFR brain concentration with a 3 mg/kg IP dose of TfRMAb-TNFR in mice was 6.5 nM compared with CNS TNF-α concentration in AD patients (6 pM) [78,79]. TfRMAb-TNFR also resulted in lower plasma exposure compared with equimolar doses of etanercept, due to peripheral TfR-mediated clearance of TfRMAb-TNFR, which further lowers the potential for systemic toxicity [80]. Overall, TfRMAb-TNFR is a BBB-penetrating TNFI that can bind to both peripheral and brain TNF-α.

### 3.2. TfRMAb-TNFR in AD Mouse Models

**Figure 2:**
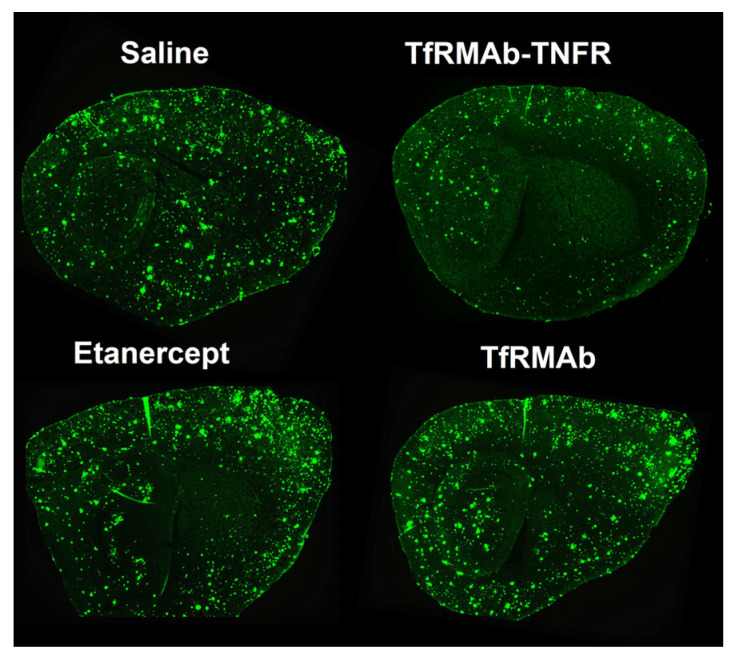
Sagittal brain sections from male APP/PS1 mice treated with saline, TfRMAb-TNFR (3 mg/kg), etanercept (1.5 mg/kg, equimolar dose), or TfRMAb (2.25 mg/kg, equimolar dose) intraperitoneally for 10 weeks show a robust reduction in 6E10-positive Aβ load with TfRMAb-TNFR. From reference [82].

TfRMAb-TNFR has demonstrated therapeutic efficacy across multiple AD mouse models. In the APP/PS1 double transgenic amyloidosis model, 6-month-old male mice treated IP with 3 mg/kg TfRMAb-TNFR for 12 weeks showed a significant reduction in brain Aβ burden (both 6E10-positive Aβ and thioflavin-S-positive mature Aβ plaques), insoluble Aβ42, neuroinflammatory markers (ICAM-1), and BBB disruption markers (parenchymal IgG), and improved recognition memory [81]. Further, chronic TfRMAb-TNFR dosing resulted in low ADA formation and no adverse immune reactions or cerebral microhemorrhages [81]. Equimolar doses of etanercept only reduced thioflavin-S-positive Aβ plaques and brain IgG and had no effect on recognition memory [81]. When treatment was delayed until 11 months of age in male APP/PS1 mice, when the Aβ load had plateaued, TfRMAb-TNFR demonstrated superior efficacy compared to non-BBB-penetrating etanercept, uniquely reducing 6E10-positive Aβ load, thioflavin-S-positive Aβ plaques, and insoluble-oligomeric Aβ (Figure 2). TfRMAb-TNFR treatment was associated with a greater accumulation of phagocytic microglia around Aβ plaques, reduced spatial memory deficits, and increased the expression of BBB tight junction proteins. These improvements were not observed with equimolar doses of etanercept. Additionally, no changes were observed in hematology indices or iron homeostasis with TfRMAb-TNFR [82].

In the PS19 tauopathy mouse model, 6-month-old male and female mice receiving TfRMAb-TNFR (1.75 mg/kg) or etanercept (0.875 mg/kg equimolar dose) for 8 weeks both showed therapeutic benefits; etanercept reduced phosphorylated tau (Ser202, Thr205) and microgliosis in both sexes, while TfRMAb-TNFR was effective in females. Both etanercept and TfRMAb-TNFR increased the expression of post-synaptic density protein 95 and attenuated neuron loss in the hippocampus despite TfRMAb-TNFR having eight-fold lower plasma exposure than etanercept [80]. In the 3xTg-AD model, which combines both amyloidosis and tauopathy, 8-month-old female mice treated with TfRMAb-TNFR (3 mg/kg IP) for 12 weeks demonstrated a predominantly microglia-centric mechanism of action [83]. TfRMAb-TNFR modulated several proteins relevant to microglial function, significantly reduced mature β-sheet-rich Aβ plaques, increased triggering receptor expressed on myeloid cell 2 (TREM2)-positive microglial clustering around plaques, and modulated proteins involved in Aβ processing (β-site APP-cleaving enzyme 1 and neprilysin), oligodendrocyte markers (myelin basic protein and oligodendrocyte transcription factor 2), disease-associated microglial markers (secreted phosphoprotein 1, purinergic receptor P2X 7, and CD163), and neurodegeneration markers (phospho-α-synuclein, TAR DNA-binding protein 43, and neurogranin). Notably, the treatment showed a favorable safety profile with minimal changes to plasma metabolic parameters [83].

Across all studies, TfRMAb-TNFR showed stable hematological parameters and low immunogenicity, and exhibited therapeutic effects through multiple complementary mechanisms, including direct Aβ and tau pathology reduction, increased phagocytic microglial phenotype, decreased neuroinflammation, BBB protection, and neuroprotection, supporting its potential as a disease-modifying therapeutic for AD.

## 4. Receptor-Mediated Brain Delivery of Anti-Aβ Antibodies

**Figure 3:**
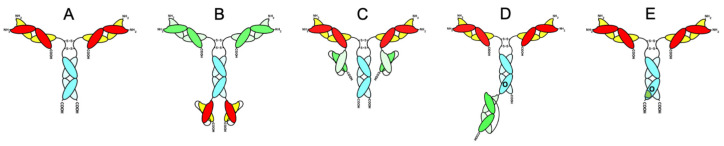
Reengineering of anti-Aβ antibodies (AAAs) as brain-penetrating bispecific antibodies (BSA). (**A**) A diagram of a conventional AAA with the Fab region in red/yellow and the constant Fc region in light blue. (**B**) TfRMAb-ScFv—this BSA is comprised by the variable domain of the rat 8D3 MAb targeting the mouse TfR fused to the mouse IgG1 and kappa regions (light green) and the mouse IgG1 Fc region (light blue). An AAA single-chain Fv (ScFv) antibody is attached at the C-terminal end (red/yellow). (**C**) A brain-penetrating AAA BSA where the transport domains are anti-TfRMAb in ScFv format (light green) fused to the C-terminus of the AAA fragment antigen-binding region, Fab (red/yellow). (**D**) A diagram of trontinemab. In this BSA, the AAA (Fab in red/yellow and Fc in light blue) is reengineered as a brain-penetrating (2 + 1) BSA, where that transport domain is a humanized anti-TfRMAb in a single-chain Fab format (light green) fused to the C-terminus of one of the two heavy chains of the AAA. (**E**) A diagram of ATV:Aβ MAb. In this brain-penetrating AAA (Fab in red/yellow and Fc in light blue) BSA, the amino acid sequence of the Fc region near the C-terminus of an IgG is modified to bind the TfR with low affinity (green dot). The heterodimer in (**D**,**E**) is engineered with the knobs-into-holes technology (O). Recreated from [13,84,86,87].

The immunotherapy of AD with antibodies directed against Aβ is a potential treatment for this disorder. Several anti-Aβ antibodies (AAAs) were approved for the treatment of AD [1,2,3,4,5,6]. However, these antibodies presented a low therapeutic index due to poor brain penetration and adverse effects like ARIA [7]. AAAs may be reengineered as brain-penetrating BSA targeting the BBB TfR. The first BBB-penetrable AAA was re-engineered as a tetravalent BSA by fusing the amino terminus of the single-chain Fv (ScFv) of a mouse AAA to the carboxyl terminus of the heavy chain of a rat/mouse chimeric monoclonal antibody against the mouse TfR (TfRMAb-ScFv; Figure 3B) [84,85]. The AAA in this case was directed towards the amino terminal of the Aβ peptide. The TfRMAb-ScFv fusion protein demonstrated bifunctionality, binding both the targets, Aβ and the mouse TfR, enabling receptor-mediated transport across the BBB. Accordingly, TfRMAb-ScFv showed high brain penetration of 3.5 ± 0.7% ID/g brain via TfR-mediated BBB transport, which is nearly 60-fold higher than that of a non-targeting antibody (0.06 ± 0.01% ID/g brain) [84].

### 4.1. TfRMAb-ScFv in AD Mouse Models

TfRMAb-ScFv was initially tested in 11-month-old male APP/PS1 mice at a dose of 1 mg/kg injected IV every 3–4 days per week over the course of 12 weeks. Chronic TfRMAb-ScFv dosing reduced insoluble brain Aβ42 by 40% [84]. However, unlike conventional AAAs with plasma half-lives of weeks, the TfRMAb-ScFv fusion protein is rapidly cleared from blood with a median residence time of 175 ± 32 min [84]. Therefore, to maintain sustained brain exposure to the AAA, in a follow-up study, TfRMAb-ScFv was injected in 12-month-old male APP/PS1 mice via the SQ route daily at a dose of 5 mg/kg for 12 weeks [88]. The new dosing regimen resulted in a significant 60% reduction in 6E10-positive Aβ load and thioflavin-S-positive Aβ plaque load. Notably, no elevation in plasma Aβ, no cerebral microhemorrhage, and only low-titer ADA responses were observed with both dosing regimens, providing the first proof-of-concept that AAAs can be successfully re-engineered as safe and effective BBB-penetrating therapeutics for AD.

### 4.2. Trontinemab

In trontinemab, the AAA gantenerumab [89] is reengineered as a brain-penetrating (2 + 1) BSA, and it represents the first biological of its class completing a phase I/II clinical trial for the immunotherapy of AD (NCT04639050) (Figure 3D) [13]. The transport domain in trontinemab is a humanized anti-TfRMAb in a single-chain fragment antigen-binding region (Fab) format fused to the C-terminus of one of the two gantenerumab heavy chains. So, this BSA binds to the TfR in monomeric form with low affinity, i.e., KD = 131 nM (Figure 3D) [13]. Phase I/II clinical trial in AD subjects showed a marked reduction in Aβ in the brain by amyloid positron emission tomography (PET) following IV administration of 1.8 mg/kg or 3.6 mg/kg trontinemab every 4 weeks for 28 weeks. AD biomarkers, including CSF Aβ and total and p-Tau, were also diminished [13,90]. Trontinemab showed a good safety profile as compared with unmodified AAA [7,13], with <5% of participants presenting signs of ARIA, including three mild cases of ARIA-E (edema) and five cases of ARIA-H (hemorrhage), with most of these effects seen with the 1.8 mg/kg dose. Follow-up data showed that >90% of individuals on the 3.6 mg/kg dose were Aβ negative, with a 1% ARIA-E rate. Data on trontinemab are encouraging, and it is currently undergoing a phase III clinical trial (NCT07169578) [90].

### 4.3. Other Constructs for the Immunotherapy of AD

Other constructs for the immunotherapy of AD were reported using the 8D3 anti-mouse TfR shuttle system [86,91]. A complete IgG of the recombinant variant of anti-Aβ mAb158 [92] is transported across the BBB by the 8D3 MAb, which is fused in a scFv format to the C-terminus of the light chain of the AAA to form a tetravalent BSA (Figure 3C) [86,91]. In a mouse model of AD, mAb158-8D3 BSA showed a marked reduction in soluble Aβ protofibrils [91]. Aβ-affibodies were also reengineered to cross the BBB by fusion to a monovalent scFv of the 8D3 MAb [93]. The anti-mouse 8D3 MAb was also used in a tetravalent bispecific tandem-Aβ construct [94]. This BSA showed low affinity for the TfR and efficacy in an AD mouse model, in the absence of serious adverse effects [94].

### 4.4. ATV:Aβ MAb

ATV stands for antibody transport vehicle, and it is produced by the modification of the amino acid sequence of the Fc region near the C-terminus of an IgG to bind the TfR with low affinity, inducing RMT across the BBB (Figure 3E). This technology was applied to an AAA to produce an asymmetrical MAb with a TfR binding in one heavy chain and Fc mutations (ATV^cisLALA^) in the other heavy chain engineered to mitigate TfR-related hematological adverse side effects (DNL921, ATV:Abeta) [87]. This construct showed complete elimination of vascular inflammation and ARIA lesions in a mouse model of AD [87].

## 5. Other Antibody-Based Biologicals for AD

Neprilysin (NEP) is a zinc-dependent metalloprotease that cleaves monomeric and pathological oligomeric Aβ40 [95]. Therefore, NEP may represent a novel treatment for AD, provided that a brain-penetrating form of NP is produced. Such a molecule was configured with the mouse anti-rat TfR OX26 Fab fused to hIgG1 constant region, and with the extracellular domain of NEP fused to the C-terminus of the OX26-Fab-Fc shuttle antibody [96]. In an animal model, dosing of the OX26-Fab-Fc-NEP fusion protein resulted in a marked reduction in Aβ40 [96]. Another brain-penetrating NEP fusion engineered with the rat anti-mouse TfR 8D3 scFv was also effective in a mouse model of AD [97].

The activity of NEP may be augmented by somatostatin (SST). A brain-penetrating form of SST was produced by fusion to the 8D3-scFv, and it was effective in increasing the endogenous levels of NEP in the brain and inducing degradation of membrane-associated Aβ42 [98]. Other potential therapies using brain shuttles to transport biologics into the brain include the oligonucleotide transport vehicle, which uses the TfRMAb to deliver an oligonucleotide targeting the MAPT gene for AD (DNL628 and NCT07328451). Additionally, a BBB-penetrating variant of an antibody activating TREM2 was engineered by fusing a high-affinity TREM2-activating antibody to an ATV. In AD mice, TREM2:ATV increased microglial response and brain glucose metabolism [99].

## 6. Discussion

The data reviewed in the present article are consistent with the following conclusions. First, EPO may be reengineered as a brain-penetrating antibody fusion protein targeting the BBB-TfR for the treatment of AD (Section 2). Second, TNFI may also be reengineered as a brain-penetrating antibody fusion protein targeting the BBB-TfR for the treatment of AD (Section 3). Third, MAbs directed to Aβ may be reengineered into a brain-penetrating antibody fusion protein targeting the BBB-TfR for the treatment of AD (Section 4, Figure 3). Fourth, targeting brain Aβ using brain-penetrating forms of NEP or SST may represent novel strategies for the treatment of AD (Section 5).

The immunotherapy of AD with antibodies directed against Aβ is possible, and conventional AAAs have been approved by regulatory agencies, i.e., aducanumab, donanemab, and lecanemab [1,2,3,4,5,6]. However, the brain penetration of these MAbs is markedly reduced by the presence of the BBB, which is only permeable to lipophilic molecules of <400 Da [8,9,10,11,12]. In addition to the marginal therapeutic efficacy of these MAbs, they were associated with adverse effects like ARIA [7], which are intrinsic to these MAbs [100]. Interestingly, reengineering of the anti-Aβ gantenerumab [89] as a brain-penetrating (2 + 1) BSA in trontinemab markedly reduced the incidence of ARIA [13]. Other anti-Aβ BSA fusion proteins based on the 8D3 MAb directed to the mouse TfR reported no evidence of brain microhemorrhage [84,85,86,88,91,93,94]. Finally, the ATV:Aβ MAb showed complete elimination of vascular inflammation and ARIA lesions in a mouse model of AD [87]. Data overall suggest that the ARIA serious adverse effects associated with the immunotherapy of AD may be eliminated by the conversion of Aβ-MAbs into brain-penetrating BSA.

Trontinemab represents the first biological of its class completing a phase I/II clinical trial for the immunotherapy of AD (NCT04639050) [13]. This brain-penetrating anti-Aβ BSA showed promising results in AD subjects with marked reduction in Aβ in the brain by PET and AD biomarkers in CSF [90]. The major side-effects reported with trontinemab were infusion-related reactions and anemia; the latter was transient. The ARIA-associated adverse effects were reduced to <5%, an effect attributed to targeting of parenchymal Aβ instead of vascular Aβ with trontinemab compared with conventional AAAs, and trontinemab is currently in phase III clinical trial with the 3.6 mg/kg dose [90].

Besides the re-engineering of AAAs for brain penetration for AD treatment, pre-clinical data indicate that EPO [31,53,58,59,60,61] and TNFI [76,78,80,81,82,83] may be re-engineered for brain penetration for AD. These brain-penetrable biologics target several pathways implicated in AD pathology, including neurodegeneration and neuroinflammation, offering a different mechanistic approach to treat AD. Other potential new treatments for AD are based on brain-penetrating NEP and SST, which are aimed at cleaving monomeric and pathological oligomeric Aβ40 [96,97,98].

Based on the data reviewed, it is possible to reengineer antibodies and protein-based biologicals into brain-penetrating forms by fusion to antibodies, antibody-fragments, and/or binding domains targeting the BBB TfR. The latter induces RMT across the BBB, increasing the brain uptake and intended pharmacological effect, and reducing adverse effects. In this format, trontinemab is the first biological of its class to complete a phase I/II clinical trial for the immunotherapy of AD with promising results. Other brain-penetrating biologicals under pre-clinical development also show potential benefits for the treatment of AD. Given the rapidly emerging developments in TfR targeting for AD drug delivery, this narrative review aims to summarize the progress made in this field to date. As is inherent to the narrative review format, no formal methodology was applied for the inclusion or exclusion of articles; rather, the relevant literature was selected based on its contribution to the presented topic.

## 7. Conclusions and Future Research Directions

The evidence gathered in this article suggests that brain-penetrating, antibody-based biologicals hold considerable promise. Pending further pre-clinical development and successful clinical trials, these brain-penetrating agents are well-positioned to emerge as a novel and transformative class of therapeutics for AD.

## Data Availability

No new data were created or analyzed in this study.

## Abbreviations

The following abbreviations are used in this manuscript:

AD	Alzheimer’s disease
AAA	anti-amyloid antibody
ADA	anti-drug antibody
ARIA	amyloid-related imaging abnormalities
ATV	antibody transport vehicle
BBB	blood–brain barrier
BSA	bispecific antibodies
CNS	central nervous system
CSF	cerebrospinal fluid
ECD	extracellular domain
EPO	erythropoietin
Fab	fragment antigen-binding region
Fc	Fragment crystallizable region
ID	injected dose
IP	intraperitoneal
IV	intravenous
KD	dissociation constant
LSD	lysosomal storage disorders
MAb	monoclonal antibody
NEP	neprilysin
RMT	receptor-mediated transcytosis
scFv	single-chain Fv
SQ	subcutaneous
SST	somatostatin
TfR	transferrin receptor
mTfR	mouse TfR
TNF-α	tumor necrosis factor alpha
TNFI	TNF inhibitor
TNFR	TNF receptor
TREM2	triggering receptor expressed on myeloid cells 2
